# Bioavailability of Organosulfur Compounds after the Ingestion of Black Garlic by Healthy Humans

**DOI:** 10.3390/antiox12040925

**Published:** 2023-04-13

**Authors:** Alicia Moreno-Ortega, Gema Pereira-Caro, Iziar A. Ludwig, María-José Motilva, José Manuel Moreno-Rojas

**Affiliations:** 1 Department of Agroindustry and Food Quality, Andalusian Institute of Agricultural and Fisheries Research and Training (IFAPA), Alameda del Obispo, Avda. Menéndez-Pidal, 14004 Córdoba, Spain; mariag.pereira@juntadeandalucia.es; 2 Foods for Health Group, Maimonides Biomedical Research Institute of Cordoba (IMIBIC), 14004 Córdoba, Spain; 3 Agrotecnio Center, XaRTA-TPV, Food Technology Department, Escola Tècnica Superior d’Enginyeria Agrària, University of Lleida, Avda. Alcalde Rovira Roure 191, 25198 Catalonia, Spain; iludwig@unav.es (I.A.L.);

**Keywords:** black garlic, bioavailability, human urine, organosulfur compounds, *N*-acetyl-*S*-allyl-L-cysteine (NASAC), alliin, HPLC-HRMS analysis

## Abstract

The consumption of black garlic has been related to a decreased risk of many human diseases due to the presence of phytochemicals such as organosulfur compounds (OSCs). However, information on the metabolization of these compounds in humans is limited. By means of ultra-high-performance liquid chromatography coupled with high-resolution mass spectrometry (UHPLC-HRMS), this study aims to determine the OSCs and their metabolites excreted in urine 24 h after an acute intake of 20 g of black garlic by healthy humans. Thirty-three OSCs were identified and quantified, methiin (17,954 ± 6040 nmol), isoalliin (15,001 ± 9241 nmol), *S*-(2-carboxypropyl)-L-cysteine (8804 ± 7220 nmol) and S-propyl-L-cysteine (deoxypropiin) (7035 ± 1392 nmol) being the main ones. Also detected were the metabolites *N*-acetyl-*S*-allyl-L-cysteine (NASAC), *N*-acetyl-*S*-allyl-L-cysteine sulfoxide (NASACS) and *N*-acetyl-*S*-(2-carboxypropyl)-L-cysteine (NACPC), derived from *S*-allyl-L-cysteine (SAC), alliin and *S*-(2-carboxypropyl)-L-cysteine, respectively. These compounds are potentially *N*-acetylated in the liver and kidney. The total excretion of OSCs 24 h after the ingestion of black garlic was 64,312 ± 26,584 nmol. A tentative metabolic pathway has been proposed for OSCs in humans.

## 1. Introduction

Garlic (*Allium sativum* L.) is a basic ingredient greatly appreciated in the world’s cuisines for its organoleptic characteristics and the health properties associated with its consumption [1]. In this sense, regular consumption of garlic has been related to a protective effect against diseases such as colorectal, lung, gastric and bladder cancer by regulating the metabolism of carcinogenic substances, inducing apoptosis or inhibition of the proliferation of cancer cells, among other mechanisms [2,3,4].

Black garlic, a garlic-derived product, has generated growing interest in recent years for its organoleptic qualities and physical and chemical characteristics. It is obtained by subjecting fresh garlic to thermal processing (70–80 °C) under controlled humidity conditions for 1–3 months [5,6,7], which induces chemical reactions in garlic, such as enzymatic browning and the Maillard reaction. This elaboration process results in a final product with a brownish colour, sweeter taste, softer texture and low pungency [8,9]. In addition, compositional changes are produced, with *S*-allyl-L-cysteine (SAC) being the predominant organosulfur compound in black garlic [10]. The described changes in the profile of phytochemical compounds during the processing of black garlic have attracted scientific interest, leading to multiple studies aimed at assessing the potential health effects of the regular consumption of black garlic [11,12,13,14]. Black garlic consumption has been associated with a decrease in blood pressure and total plasma cholesterol, both of which are risk factors for cardiovascular disease [15,16,17,18]. Similarly, the consumption of this product has been related to a protective effect against other medical conditions such as neurodegenerative, digestive or renal diseases [12,19,20,21]. These health properties associated with its consumption are mainly due to the presence of compounds with recognised bioactivity, particularly organosulfur compounds [11]. However, the mechanisms by which organosulfur compounds in black garlic may exert these positive effects on health need to be further elucidated. It is necessary to know their metabolism and bioavailability in order to gain a deeper understanding of their bioactive potential.

Previous in vitro studies have provided information on the bioaccessibility of phytochemical compounds in black garlic, as well as on the evolution of these compounds during fermentation at the colonic level [22]. Regarding the bioavailability of organosulfur compounds, some studies have been carried out in fresh garlic, but assessing mainly those of a volatile nature [23,24,25,26,27]. Furthermore, the bioavailability of isolated organosulfur compounds in black garlic has been evaluated, such as the excretion of SAC and its *N*-acetylated form in the urine of rats, mice and dogs, and the metabolization of cycloalliin in rats [28,29]. However, to the best of our knowledge, there are no studies evaluating the bioavailability of organosulfur compounds after ingestion of black garlic by humans.

Based on these observations, the aim of the present study was to identify and quantify the organosulfur compounds excreted in urine 24 h after the acute intake of 20 g of black garlic in humans. To study the organosulfur biological metabolites, a targeted chromatographic approach based on ultra-high performance liquid chromatography coupled with high-resolution mass spectrometry (UHPLC-HRMS) was used.

## 2. Materials and Methods

### 2.1. Chemicals

The reference standard compounds including *S*-allyl-L-cysteine sulfoxide (alliin) and *S*-allyl-L-cysteine (SAC) were purchased from Sigma-Aldrich (Madrid, Spain). Ammonium acetate, ammonium formate and ethanol were obtained from Sigma-Aldrich. Acetonitrile, methanol and water were of LC-MS grade.

### 2.2. Study Design

Twelve healthy volunteers (seven females and five males, mean age 29.0 ± 8.9 years) with a body mass index (BMI) of 23.5 ± 2.2 kg/m^2^ (mean ± SD) participated in this study. Exclusion criteria were pregnancy or lactation, drug allergies, any chronic medication and any antibiotic treatment during the 6 months prior to the study. Written informed consent was provided by all participants. The study protocol was approved by the Ethical Committee of the Human Clinical Research Unit at the Arnau Vilanova University Hospital, Lleida, Spain (Approval Number: CEiC-1790).

Before attending the clinic, participants were asked to follow a diet free of organosulfur compounds for two days by avoiding allium vegetables. On the morning of the feeding trial, the volunteers brought their 12 h overnight urine (basal) and were invited to eat a portion of 20 g of black garlic after fasting overnight and to continue with the organosulfur-free diet that day and until the next morning, when the last urine sample was collected. Urine samples were obtained at interval times of 0–2, 2–4, 4–8 and 8–24 h after the black garlic intake. The total volume of each sample was recorded before storing the aliquots (2 mL) at −80 °C until the chromatographic analysis.

### 2.3. Processing of Urine Samples

The urine samples were defrosted, vortexed and centrifuged at 15,000 rpm for 10 min at 4 °C and the supernatant was collected and stored at −80 °C until UHPLC-HRMS analysis. Each sample was prepared in duplicate.

### 2.4. Chromatographic Analysis of Organosulfur Compounds in Urine Samples

Aliquots of human urine samples were analysed using a UHPLC-PDA-MS mass spectrometer system (Thermo Scientific, San José, CA, USA) comprising a UHPLC pump, a PDA detector scanning from 200 to 600 nm and an autosampler operating at 4 °C (Dionex Ultimate 3000 RS, Thermo Corporation, San José, CA, USA). The chromatographic conditions were previously described by Moreno-Rojas et al. (2018) [30]. Briefly, organosulfur compounds were separated on a 2.1 × 150 mm ACQUITY UPLC 1.7 μm BEH amide column (equipped with an ACQUITY UPLC BEH amide 1.7 μm van-guard pre-column) (Waters, Spain), which was maintained at 35 °C and eluted at a flow rate of 0.4 mL/min with a 20 min gradient using two mobile phases: A: deionized water with 5 mM of ammonium acetate, 5 mM ammonium formate and 1% formic acid; and B: acetonitrile. The gradient started with 5% of A rising to 46% in 13 min and finally returning to 5% of A and was maintained for 4 min to equilibrate the column.

After passing through the flow cell of the PDA detector, the column eluate was directed to an Exactive Orbitrap mass spectrometer (Thermo Scientific, San José, CA, USA) fitted with a Heated Electrospray Ionization Probe (HESI) operating in positive ionization mode for the determination of organosulfur compounds [30]. Data acquisition and processing were carried out using the Xcalibur 3.0 software (Thermo Scientific, San José, CA, USA).

The organosulfur compounds in the human urine samples were identified by comparing the exact mass and the retention time with available standards. In the absence of standards, compounds were tentatively identified by comparing the exact theoretical mass of the molecular ion with the measured accurate mass of the molecular ion and searching against metabolite databases, including Metlin, Human Metabolome DataBase (HMDB) and more general chemical databases, such as PubChem and ChemSpider. The organosulfur compounds were quantified by selecting the exact theoretical mass of the molecular ion by reference to standard curves, obtaining a linear regression analysis with R^2^ values of >0.998 (*n* = 6) and an equation of the line with a slope of 427,961 and an intersection of −169,143 for the alliin, and with a slope of 869,774 and an intersection of −232,084 for the SAC. The detection and quantification limits were 0.03 and 0.1 ng/μL, respectively. In the absence of reference compounds, they were tentatively quantified by reference to the calibration curve of a closely related parent compound.

### 2.5. Statistical Analysis

A one-way ANOVA was carried out using R software (v.3.6.3., R Core Team, Vienna, Austria) to determine the overall differences between the human urine samples at different interval times after the intake of black garlic If significance was detected, a Fisher’s LSD post hoc test was then performed to compare the different interval times. A *p*-value < 0.05 was considered statistically significant. In addition, a Pearson correlation (*p* < 0.05) analysis was performed by the software SIMCA v. 17 (Umetrics, Umea, Sweden).

## 3. Results and Discussion

### 3.1. Characterisation of Organosulfur Compounds in Black Garlic

**Figure 1:**
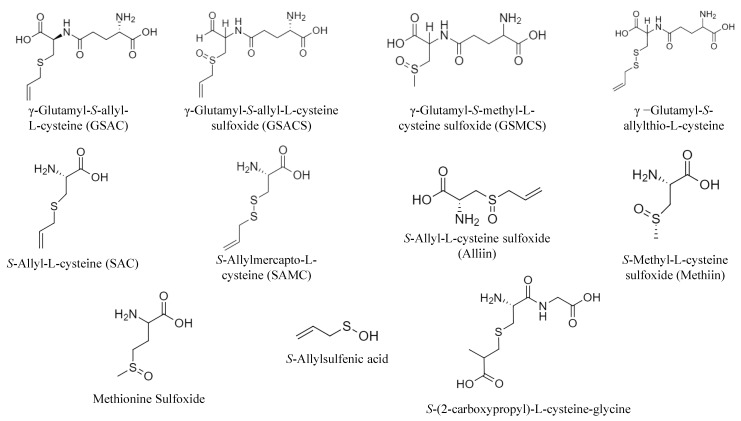
Chemical structures of organosulfur compounds identified in black garlic in more than 1% of the total organosulfur compounds.

The UHPLC-HRMS analysis of the black garlic fed to the volunteers revealed the presence of twenty organosulfur compounds, in agreement with our previous study [10] (Table 1). A total of 510 µmol of organosulfur compounds was present in the 20 g of black garlic ingested by the volunteers, of which 187 µmol corresponded to γ-glutamyl-*S*-alk(en)yl-L-cysteine derivatives (≈37% of the total organosulfur content) and 323 µmol to *S*-alk(en)yl-L-cysteine derivatives (≈63% of the total). Particularly, the compounds *S*-allyl-L-cysteine (SAC) (154 µmol), alliin (112 µmol), γ-glutamyl-*S*-allyl-L-cysteine (GSAC) (87.9 µmol) and γ-glutamyl-*S*-allyl-L-cysteine sulfoxide (GSACS) (69.0 µmol) were the main organosulfur compounds determined in 20 g of black garlic, accounting for 83% of the total organosulfur content. These were followed by S-allylsulfenic acid, γ-glutamyl-*S*-allylthio-L-cysteine and methionine sulfoxide, with 18.70, 10.04 and 10.08 µmol, respectively. Small quantities of other organosulfur compounds, such as *S*-allylmercapto-L-cysteine (8.16 µmol), γ-glutamyl-*S*-methyl-L-cysteine sulfoxide (7.60 µmol), *S*-(2-carboxypropyl)-L-cysteine-glycine (5.2 µmol) and methiin (4.86 µmol) were also present. According to these data, Ríos-Ríos et al. (2019) [8] also reported that SAC and alliin were the main organosulfur compounds found in black garlic. SAC is mainly produced by the action of γ-glutamyl transpeptidase (γ-GTP) hydrolysing GSAC [31]. While alliin, one of the major compounds in fresh garlic, is hydrolysed by alliinase enzymes, its content in black garlic remains high as alliinase enzymes are usually inactivated during the processing of black garlic [32,33]. Figure 1 shows the chemical structures of the main organosulfur compounds found in black garlic.

### 3.2. Identification and Quantification of Organosulfur Compounds and their Metabolites in Urine

After the acute ingestion of 20 g of black garlic by the 12 volunteers, urine collected over 24 h was analysed by HPLC-HRMS, and thirty-three organosulfur compounds were identified. Details of the retention time, chemical formula, experimental mass and identification are presented in Table 2, and their chromatograms in Figures S1 and S2. Of the 33 compounds identified in urine, 12 were GSAk derivatives and 21 were SAk derivatives. Table 3 shows the urinary excretion of the organosulfur compounds determined 0–24 h after black garlic consumption. None of these compounds were found in urine 12 h prior to the intake of black garlic. Moreover, except for two GSAk derivatives (γ-glutamyl-*S*-(2-carboxyethyl)-L-cysteine-glycine, γ-glutamyl-S-allylthio-L-cysteine) and four SAk derivatives (deoxymethiin, *S*-allylmercapto-L-cysteine, *S*-propylmercapto-L-cysteine and *S*-allylsulfenic acid) that were not detected in the urine, the remaining parent organosulfur compounds present in black garlic were detected in the urine after the ingestion of this product. The total urine excretion of organosulfur compounds 0–24 h after the ingestion of black garlic was 64.312 ± 26.584 nmol, equivalent to 12.9% of the 498.8 µmol of organosulfur compounds ingested in the 20 g of black garlic (Table 3), with 97.3% of these being SAk derivatives and 2.7% GSAk derivatives. The bioavailability of isolated compounds characteristic of garlic, such as SAC, has been evaluated previously by other authors, obtaining bioavailability percentages between 87 and 103% [28]. However, a low bioavailability of garlic OSCs, such as allicin, has also been reported, probably because they are bound by other compounds such as proteins or fatty acids [34,35]. The low percentage of organosulfur compounds excreted in urine in the present study might also be due to the formation of intermediate metabolites and/or volatile compounds which could not be determined by targeted chromatography [27].

The main organosulfur compounds determined in the human urine were methiin (17,953 ± 6040 nmol), isoalliin (15,001 ± 9241 nmol), *S*-(2-carboxypropyl)-L-cysteine (8804 ± 7220 nmol) and *S*-propyl-L-cysteine (deoxypropiin) (7035 ± 1392 nmol), accounting for 75.9% of the total organosulfur compounds excreted after the intake of black garlic. A substantial amount of SAC (2991 ± 1059 nmol), tran-*S*-(1-propenyl)-L-cysteine (S1PC) (3911 ± 987 nmol), methionine sulfoxide (2221 ± 343 nmol) and alliin (1383 ± 1035 nmol) were also quantified. These results are in keeping with previous studies on black garlic, where alliin and SAC were detected after simulated gastrointestinal digestion, and methionine sulfoxide, SAC and propiin were detected after colonic fermentation in vitro [10,22]. Regarding the high concentration of isoalliin quantified, several studies have reported that during both the gastrointestinal digestion and biological metabolism of these compounds, isomerization reactions may take place, possibly resulting in an increased concentration of isoalliin derived from alliin metabolism [36,37,38].

**Figure 2:**
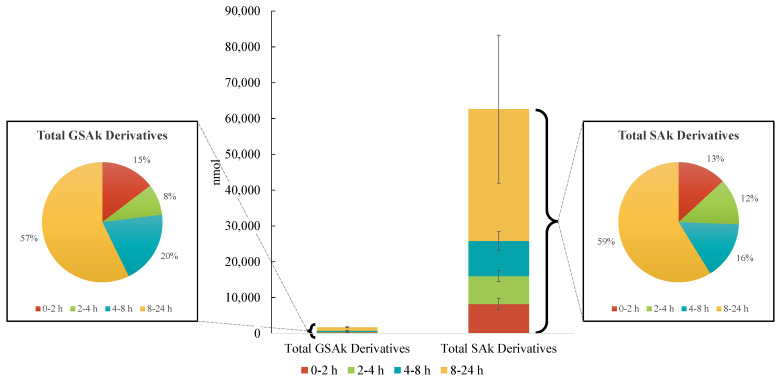
Time-course profiles of the organosulfur compounds identified during the urinary excretion after the intake of black garlic. GSAk: γ-Glutamyl-*S*-alk(en)yl-L-cysteine; SAk: *S*-alk(en)yl-L-cysteine.

Regarding the total organosulfur compounds, 59% were excreted between 8 and 24 h after the ingestion of black garlic, which likely means that the absorption of these compounds mainly occurs at the level of the large intestine (Figure 2).

Among the organosulfur compounds detected in the 24 h urine, *N*-acetyl-*S*-allyl-L-cysteine (NASAC), *N*-acetyl-*S*-allyl-L-cysteine sulfoxide (NASACS) and *N*-acetyl-*S*-(2-carboxypropyl)-L-cysteine (NACPC) are the *N*-acetylated derivatives of alliin, SAC and *S*-(2-carboxypropyl)-L-cysteine, respectively. NASAC and NASACS have previously been described as urinary biomarkers of garlic consumption and are considered to be metabolites of SAC. This was observed by Nagae et al. [28], who administered SAC orally and intravenously to rats, mice and dogs and subsequently determined the concentration of this compound and its metabolites (NASAC and NASACS) in plasma, urine, bile and different organs. Similarly, a study by Verhagen et al. [39] in humans detected NASAC in the urine of volunteers with a garlic-supplemented diet, but not in the urine of the control diet volunteers. Different studies have revealed a complex metabolism of the organosulfur compounds in garlic. Amano et al. [40] showed that *N*-acetylation of these compounds occurred in the liver and kidney by amino acid transporters located in the intestinal lumen of mammalian species [41,42]. In this sense, they evaluated the *N*-acetylation activity of the liver and kidney of rats and dogs, observing that *N*-acetylation occurred to a greater extent in rats, while in dogs, the kidney showed significantly higher NASACS deacetylation activity than *N*-acetylation. The results obtained in our study suggest that significant deacetylation activity can also be produced in the human liver and kidney, since the concentration of SAC (2991 ± 1059 nmol) found in the urine after 24 h was significantly higher than that of NASAC (23 ± 3 nmol) and NASACS (1079 ± 512 nmol). A possible explanation for this is that amino acids are actively reabsorbed from the urine via several types of transporters in the kidney [42,43]. Moreover, Krause et al. [44] evaluated the chemometrics of SAC metabolization, reporting a higher rate of sulfoxidation of SAC to alliin than *N*-acetylation of SAC to NASAC. Additionally, possibly because of this, alliin was found in higher concentrations in the urine samples than its metabolites NASAC and NASACS.

Previous studies have reported that garlic intake reduces the amount of *N*-nitrosoproline (NPRO) excreted, probably related to the presence of NASAC in urine. NPRO formation is used as a biomarker to monitor in vivo nitrosation capacity, a process that has been related to the formation of carcinogenic nitrosamines [45]. Additionally, SAC could be present in urine due to the degradation of more complex molecules such as GSAC, GSACS, alliin or *S*-(2-carboxypropyl)-L-cysteine, as described by Yamaguchi et al., 2020 [46].

Moreover, Praticò et al. [47] reported a human study in which a significant increase in urinary *N*-acetyl-*S*-(2-carboxypropyl)-L-cysteine (NACPC) content was observed after the ingestion of garlic and onion. This compound, considered a biomarker of the consumption of *Allium* vegetables, is formed by the *N*-acetylation of *S*-(2-carboxypropyl)-L-cysteine.

**Figure 3:**
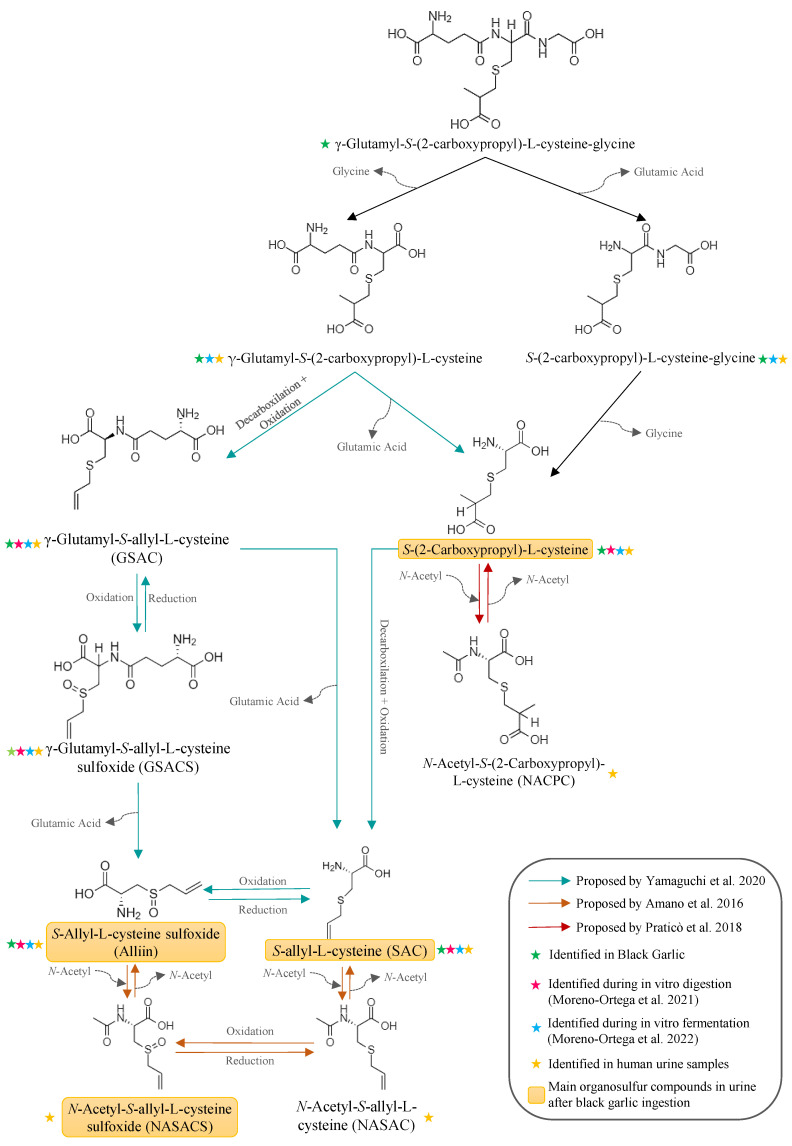
Potential pathway for the metabolism of γ-glutamyl-*S*-(2-carboxypropyl)-L-cysteine-glycine in the gastrointestinal tract after black garlic consumption. The proposed metabolism is in keeping with the findings of Yamaguchi et al., 2020, Amano et al., 2015 and Praticò et al., 2018, who monitored the metabolism of SAC in animal species. Boxed compounds indicate the main components excreted in urine after black garlic intake [40,46,47].

In view of all the information available in the literature and from our results, a potential pathway for the in vivo metabolism of organosulfur compounds in humans is summarized in Figure 3. The parent compound γ-glutamyl-*S*-(2-carboxypropyl)-L-cysteine-glycine, present in black garlic, can lose glutamic acid or glycine after hydrolysis, forming *S*-(2-carboxypropyl)-L-cysteine-glycine or γ-glutamyl-*S*-(2-carboxypropyl)-L-cysteine, respectively. Both compounds could potentially be degraded by hydrolysis to *S*-(2-carboxypropyl)-L-cysteine, one of the main organosulfur compounds found in urine and a precursor of NACPC, via the *N*-acetylation reaction.

**Figure 4:**
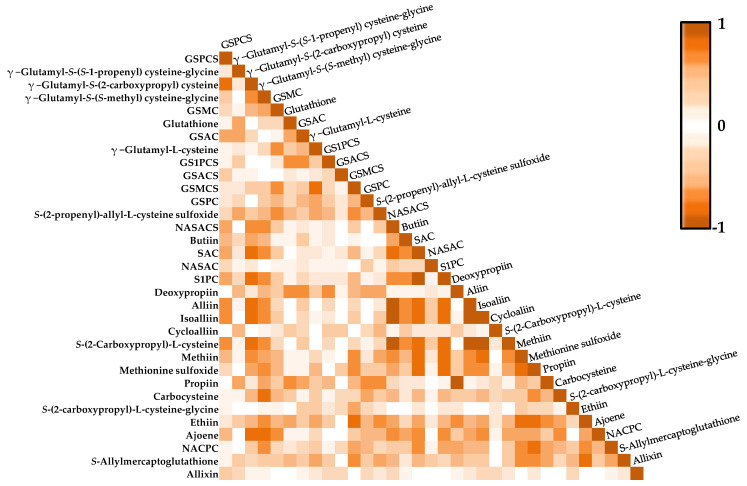
Correlation Heat Map based on Pearson correlation (*p* < 0.05). The colour indicates the level of correlation: dark orange indicates a strong correlation between the compounds and white indicates no correlation. GSPCS: γ-Glutamyl-*S*-(propyl)-L-cysteine sulfoxide; GSMC: γ-Glutamyl-*S*-methyl-L-cysteine; GSAC: γ-Glutamyl-*S*-allyl-L-cysteine; GS1PCS: γ-Glutamyl-*S*-(1-propenyl)-L-cysteine sulfoxide; GSACS: γ-Glutamyl-*S*-allyl-L-cysteine sulfoxide: GSMCS: γ-Glutamyl-*S*-methyl-L-cysteine sulfoxide; GSPC: γ-Glutamyl-*S*-(propyl)-L-cysteine; NASACS: *N*-Acetyl-*S*-allyl-L-cysteine sulfoxide; SAC: *S*-Allyl-L-cysteine; NASAC: *N*-Acetyl-*S*-allyl-L-cysteine; S1PC: trans-*S*-(1-Propenyl)-L-cysteine; NACPC: *N*-Acetyl-*S*-(2-carboxypropyl)-L-cysteine;.

The Pearson correlation test was performed in the 24 h excretion of the organosulfur compounds after the acute intake of black garlic. The correlation heatmap (Figure 4) showed a strong relationship among most of the organosulfur compounds belonging to the same putative metabolic pathways proposed (Figure 4). The colour indicates the level of correlation: dark orange indicates a strong correlation between the compounds and white indicates no correlation.

According to the metabolic pathway proposed in Figure 3, alliin showed a high correlation with some of its precursors such as γ-glutamyl-*S*-(2-carboxypropyl)-L-cysteine and *S*-(2-carboxypropyl)-L-cysteine, as well as with some compounds derived from it such as SAC or the *N*-acetylated metabolite NASACS.

**Figure 5:**
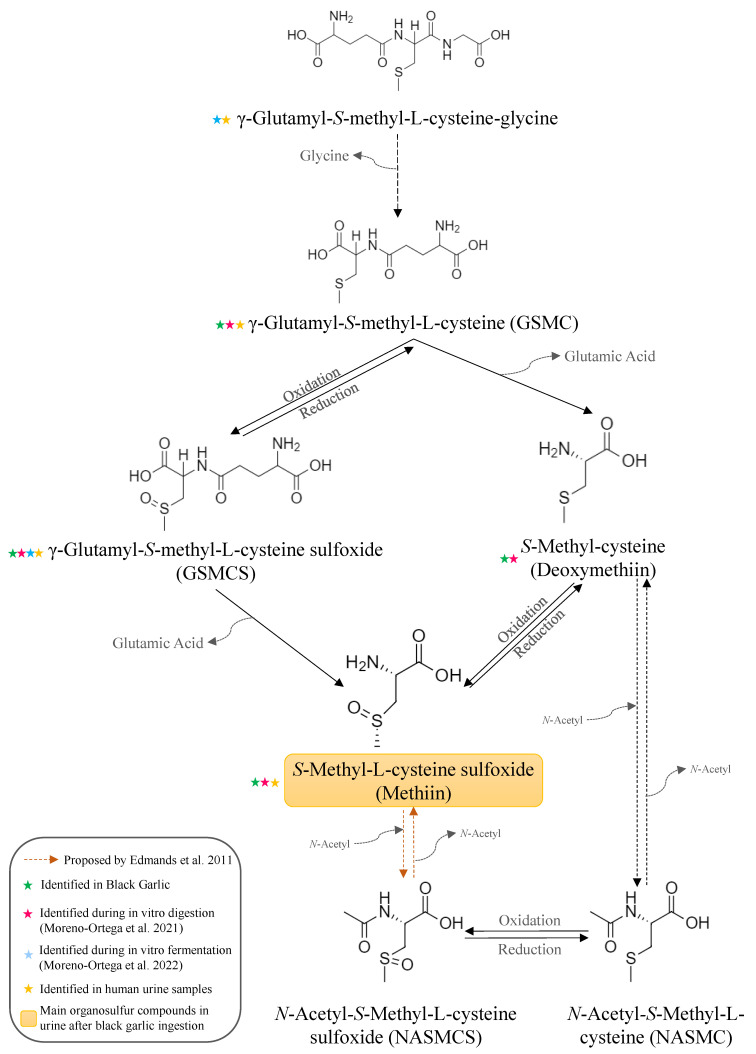
Degradation pathway proposed of γ-Glutamyl-*S*-methyl-L-cysteine-glycine based on the reports of Edmands et al., 2011 [48]. Boxes with compounds indicate the main components excreted in urine after black garlic intake.

**Figure 6:**
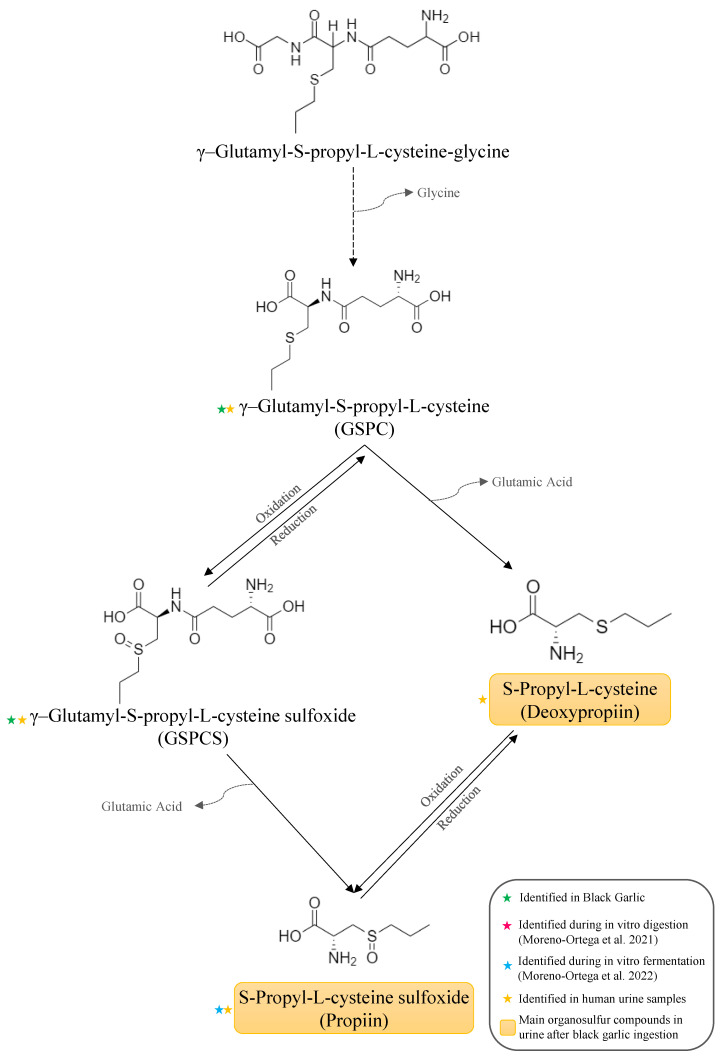
Degradation pathway proposed for γ-glutamyl-*S*-propyl cysteine-glycine. Boxes with compounds indicate the main components excreted in urine after black garlic intake.

Similarly, potential degradation pathways for other organosulfur compounds have been proposed as leading to the formation of major compounds excreted in urine such as methiin and deoxypropiin (Figure 5 and Figure 6). Methiin could be formed from the oxidation of GSMC to GSMCS and subsequent hydrolysis releasing glutamic acid, or via the elimination of glutamic acid from GSMC to obtain deoxymethiin and subsequent oxidation (Figure 5). Furthermore, methiin could also be *N*-acetylated, resulting in *N*-acetyl-*S*-methyl-L-cysteine sulfoxide (NASMCS), previously determined in human urine after the consumption of cruciferous vegetables [48], although this compound was not detected in our study. Indeed, methiin and NASCMCS can also be considered biomarkers of allium and cruciferous vegetable consumption by their presence in urine [48].

Moreover, regarding this proposed degradation pathway for γ-glutamyl-*S*-methyl-L-cysteine-glycine, the heat map (Figure 4) showed a high correlation with its derivatives: methiin, γ-glutamyl-*S*-methyl-L-cysteine (GSMC) and γ-glutamyl-*S*-methyl-L-cysteine sulfoxide (GSMCS).

Regarding the significant concentration of deoxypropiin detected in urine, it could be formed after the hydrolysis of γ-glutamyl-*S*-propyl-L-cysteine-glycine, which releases glycine and γ-glutamyl-*S*-propyl cysteine (GSPC). GSPC can be oxidised to γ-glutamyl-*S*-propyl-L-cysteine sulfoxide (GSPCS) or hydrolysed to *S*-propyl-L-cysteine (deoxypropiin). Both compounds are precursors of *S*-propyl-L-cysteine sulfoxide (propiin), coming from the hydrolysis of GSPCS and oxidation of deoxypropiin (Figure 6). It is possible that due to the chemical structures of these molecules, N-acetylation reactions of propiin and deoxypropiin could take place. Nevertheless, these metabolic pathways have not been described to date and these compounds have not been found in urine samples from the present study.

Furthermore, the compounds of the putative metabolic pathway in Figure 6 have also shown a high correlation, with deoxypropiin and propiin showing the highest correlation among them, and both showing a significant correlation with its precursor γ-Glutamyl-*S*-(propyl)-L-cysteine (GSPC).

Finally, methiin and *S*-(2-carboxypropyl)-L-cysteine showed the highest excretion percentages, accounting for 369.4 and 382.8%, respectively, in relation to the ingested garlic (20 g). This high urine excretion could probably be due to the hydrolysis of precursors of these compounds present in black garlic. In this way, degradation pathways have been proposed in an attempt to explain the obtained results (Figure 3 and Figure 5).

Several studies have related the presence of some of these organosulfur compounds with a potentially beneficial health effect. Alliin has shown antioxidant potential, decreasing or suppressing oxidative stress in rats against liver damage caused by carbon tetrachloride (CCl_4_) [49].

SAC is also a main organosulfur compound with high biological potential. Several studies have reported that SAC may act as a potential protective agent against neurodegeneration [12]. In a study on Parkinson’s disease, continuous administration of SAC has been shown to significantly decrease lipid peroxidation and the production of reactive oxygen species (ROS) [50]. Furthermore, Ashafaq et al. [51] observed that SAC attenuated oxidative damage and improved neurological deficits in rats with focal cerebral ischaemia.

On the other hand, evidence of potential biological activity of methiin as an antihyperlipidemic, antidiabetic, antimicrobial or antigenotoxic agent has been reported in animal models [52,53]. However, studies reporting on the anticancer potential of methiin are limited, and research is needed to establish the biological activity of this compound in human in vivo studies in the context of diet and disease. The identification of methiin as a urinary biomarker of black garlic consumption may provide an opportunity to further study the anti-cancer potential of this plant secondary metabolite in humans.

Nevertheless, the information available on the physiological transformations of these compounds is still very limited. Fortunately, the identification of these urinary metabolites of organosulfur compounds after the ingestion of black garlic can encourage future studies into the role of this product in human health and further the understanding of the metabolism, tissue distribution and excretion of these compounds.

## 4. Conclusions

Considering the limited information available, this study aims to identify and quantify the organosulfur compounds excreted in human urine 24 h after the acute intake of black garlic. In total, the excretion of organosulfur compounds 24 h after the ingestion of black garlic was 64,312 ± 26,584 nmol, equivalent to 12.9% of the 498.8 µmol intake of these compounds. More specifically, they consisted of 97.3% SAk derivatives and 2.7% GSAk derivatives. Thirty-three organosulfur compounds were identified and quantified in urine 24 h after ingestion, the predominant ones being methiin (17,954 ± 6040 nmol), isoalliin (15,001 ± 9241 nmol), *S*-(2-carboxypropyl)-L-cysteine (8804 ± 7220 nmol) and *S*-propyl-L-cysteine (deoxypropiin) (7035 ± 1392 nmol). Additionally, *N*-acetyl-*S*-allyl-L-cysteine (NASAC), *N*-acetyl-*S*-allyl-L-cysteine sulfoxide (NASACS) and *N*-acetyl-*S*-(2-carboxypropyl)-L-cysteine (NACPC) were detected in the urine samples, these being recognised metabolites of *S*-allyl-L-cysteine (SAC), alliin and *S*-(2-carboxypropyl)-L-cysteine, respectively. These compounds are potentially *N*-acetylated in the liver and kidney, and metabolic degradation pathways have been proposed to explain the presence of the major compounds in urine. The identification of these organosulfur compounds as urinary biomarkers of black garlic ingestion provides opportunities to study the role of this product in human health and also forms the basis for the further evaluation of the biological role and health potential of these secondary plant metabolites in humans.

## Figures and Tables

**Table 1:** Organosulfur compounds identified and quantified in 20 g of black garlic expressed as mean ± SD.

Black Garlic	µmol	% in Black Garlic
**γ-Glutamyl-*S*-alk(en)yl-L-cysteine derivatives (GSAk)**		
γ–Glutamyl-*S*-propyl-L-cysteine sulfoxide (GSPCS)	0.86 ± 0.07	0.2
γ-Glutamyl-*S*-(2-carboxypropyl)-L-cysteine	1.00 ± 0.07	0.2
γ-Glutamyl-*S*-methyl-L-cysteine (GSMC)	3.98 ± 0.00	0.8
γ-Glutamyl-*S*-allyl-L-cysteine (GSAC)	87.9 ± 0.4	17.2
γ-Glutamyl-*S*-allyl-L-cysteine sulfoxide (GSACS)	69.0 ± 0.3	13.5
γ-Glutamyl-*S*-methyl-L-cysteine sulfoxide (GSMCS)	7.60 ± 0.02	1.5
γ–Glutamyl-*S*-propyl-L-cysteine (GSPC)	0.90 ± 0.08	0.2
γ-Glutamyl-*S*-(2-carboxyethyl)-L-cysteine-glycine	2.38 ± 0.01	0.5
γ-Glutamyl-*S*-allylthio-L-cysteine	10.08 ± 0.02	2.0
γ-Glutamyl-*S*-(2-carboxypropyl)-L-cysteine-glycine	3.03 ± 0.21	0.6
**Total GSAk**	**186.7 ± 0.7**	**36.6**
***S*-alk(en)yl-L-cysteine derivatives (SAk)**		
*S*-Allyl-L-cysteine (SAC)	153.7 ± 0.3	30.1
*S*-Allyl-L-cysteine sulfoxide (Alliin)	112.2 ± 0.6	22
*S*-(2-Carboxypropyl)-L-cysteine	2.30 ± 0.00	0.5
*S*-Methyl-L-cysteine sulfoxide (Methiin)	4.86 ± 0.03	1.0
Methionine sulfoxide	10.04 ± 0.03	2.0
*S*-(2-carboxypropyl)-L-cysteine-glycine	5.20 ± 0.22	1.0
*S*-Methyl-L-cysteine (Deoxymethiin)	3.36 ± 0.02	0.7
*S*-Allylmercapto-L-cysteine (SAMC)	8.16 ± 0.02	1.6
*S*-Propylmercapto-L-cysteine (SPMC)	4.58 ± 0.01	0.9
*S*-Allylsulfenic acid	18.70 ± 0.05	3.7
**Total SAk**	**323.1 ± 0.9**	**63.4**
**Total Organosulfur Compounds**	**509.8 ± 1.6**	**100**

**Table 2:** UHPLC-HRMS-based identification of organosulfur compounds detected in human urine samples after acute intake of 20 g of black garlic.

Peak	Retention Time (min)	Compound	Chemical Formula [*m*/*z*]^-^	Experimental Mass [*m*/*z*]^-^	δ (ppm)
		**γ-Glutamyl-*S*-alk(en)yl-L-cysteine derivatives (GSAk)**			
1	4.34	γ-Glutamyl-*S*-(propyl)-L-cysteine sulfoxide (GSPCS)	C11H21N2O6S	309.1115	−2.1191
2	6.34	γ-Glutamyl-*S*-(*S*-1-propenyl)-L-cysteine-glycine	C13H22N3O6S2	380.0944	0.8362
3	6.43	γ-Glutamyl-*S*-(2-carboxypropyl)-L-cysteine	C12H19N2O7S^−2^	335.0907	−0.0579
4	7.86	γ-Glutamyl-*S*-(*S*-methyl)-L-cysteine-glycine	C11H20N3O6S2	354.0788	0.6916
5	7.88	γ-Glutamyl-*S*-methyl-L-cysteine (GSMC)	C9H17N2O5S	265.0853	0.1777
6	8.53	Glutathione	C10H18N3O6S	308.0911	0.7967
7	8.55	γ-Glutamyl-*S*-allyl-L-cysteine (GSAC)	C11H19N2O5S	291.1009	0.4285
8	8.71	γ-Glutamyl-L-cysteine	C8H15N2O5S	251.0696	−0.9444
9	8.75	γ-Glutamyl-*S*-(1-propenyl)-L-cysteine sulfoxide (GS1PCS)	C11H19N2O6S	307.0958	−0.4247
10	9.00	γ-Glutamyl-*S*-allyl-L-cysteine sulfoxide (GSACS)	C11H19N2O6S	307.0958	−1.0128
11	9.61	γ-Glutamyl-*S*-methyl-L-cysteine sulfoxide (GSMCS)	C9H17N2O6S	281.0801	−0.8092
12	9.63	γ-Glutamyl-*S*-(propyl)-L-cysteine (GSPC)	C11H21N2O5S	293.1165	−0.3883
		***S*** **-alk(en)yl-L-cysteine derivatives (SAk)**			
13	1.39	*S*-(2-Propenyl)-allyl-L-cysteine sulfoxide	C9H16NO3S	218.0845	−1.8770
14	3.92	*N*-Acetyl-*S*-allyl-L-cysteine sulfoxide (NASACS)	C8H14NO4S	220.0638	−1.2877
15	3.96	*S*-Butanoyl-L-cysteine sulfoxide (Butiin)	C7H16NO3S	194.0845	−0.8589
16	4.00	*S*-Allyl-L-cysteine (SAC)	C6H12NO2S	162.0583	−1.0605
17	4.21	*N*-Acetyl-*S*-allyl-L-cysteine (NASAC)	C8H14NO3S	204.0689	−1.9576
18	4.97	trans-*S*-(1-Propenyl)-L-cysteine (S1PC)	C6H12NO2S	162.0583	−0.5898
19	5.60	*S*-Propyl-L-cysteine (Deoxypropiin)	C6H14NO2S	164.0740	−4.2089
20	6.18	*S*-Allyl-L-cysteine sulfoxide (Alliin)	C6H12NO3S	178.0532	1.5374
21	6.70	*S*-(1-Propenyl)-L-cysteine sulfoxide (Isoalliin)	C6H12NO3S	178.0532	2.5793
22	7.10	Cycloalliin	C6H12NO3S	178.0532	2.3222
23	7.13	*S*-(2-Carboxypropyl)-L-cysteine	C7H14NO4S	208.0638	1.7404
24	7.26	*S*-Methyl-L-cysteine sulfoxide (Methiin)	C4H10NO3S	152.0376	3.5869
25	7.56	Methionine sulfoxide	C5H12NO3S	166.0532	−0.6264
26	7.87	*S*-Propyl-L-cysteine sulfoxide (Propiin)	C6H14NO3S	180.0689	2.4960
27	8.09	*S*-Carboxymethyl-L-cysteine (Carbocysteine)	C5H10NO4S	180.0325	2.5441
28	8.43	*S*-(2-Carboxypropyl)-L-cysteine-glycine	C9H17N2O5S	265.0853	0.6382
29	8.55	*S*-Ethyl-L-cysteine sulfoxide (Ethiin)	C5H12NO3S	166.0532	−0.0752
30	9.50	Ajoene	C9H15OS3	235.0280	−1.1224
31	9.58	*N*-Acetyl-*S*-(2-carboxypropyl)-L-cysteine (NACPC)	C9H16NO5S	250.0744	2.5686
32	9.74	*S*-Allylmercaptoglutathione	C13H22N3O6S2	380.0945	0.2443
33	10.89	Allixin	C12H19O4	227.1278	−1.6378

**Table 3:** Time-course profiles of the organosulfur compounds identified during urinary excretion after the intake of black garlic.

Peak	Compounds (nmol)	0–2 h	2–4 h	4–8 h	8–24 h	0–24 h	% Excretion
	**γ-Glutamyl-*S*-alk(en)yl-L-cysteine derivatives (GSAk)**						
1	γ-Glutamyl-*S*-(propyl)-L-cysteine sulfoxide	99 ± 0 a	48 ± 12 a	87 ± 19 a	88 ± 28 a	323 ± 59	37.6
2	γ-Glutamyl-*S*-(*S*-1-propenyl)-L-cysteine-glycine	1.6 ± 0.2 c	1.0 ± 0.0 bc	6.9 ± 0.0 a	2.5 ± 0.0 b	12.1 ± 0.2	-
3	γ-Glutamyl-*S*-(2-carboxypropyl)-L-cysteine	15 ± 3 a	10 ± 3 a	14 ± 2 a	27 ± 11 a	66 ± 19	6.6
4	γ-Glutamyl-*S*-(*S*-methyl)-L-cysteine-glycine	29 ± 4 b	22 ± 4 b	47 ± 7 b	157 ± 25 a	255 ± 40	-
5	γ-Glutamyl-*S*-methyl-L-cysteine (GSMC)	60 ± 11 b	37 ± 4 b	101 ± 24 b	371 ± 65 a	568 ± 104	14.3
6	Glutathione	21 ± 3 b	10 ± 2 b	31 ± 5 ab	60 ± 13 a	122 ± 22	-
7	γ-Glutamyl-*S*-allyl-L-cysteine (GSAC)	19 ± 5 b	11 ± 2 b	17 ± 4 b	42 ± 9 a	89 ± 20	0.1
8	γ-Glutamyl-L-cysteine	37 ± 8 b	18 ± 3 b	41 ± 11 ab	89 ± 18 a	185 ± 40	-
9	γ-Glutamyl S-(1-propenyl)-L-cysteine sulfoxide (GS1PCS)	3.1 ± 0.4 a	1.1 ± 0.3 a	1.7 ± 0.2 a	3.2 ± 0.1 a	9.1 ± 1.0	-
10	γ-Glutamyl-*S*-allyl-L-cysteine sulfoxide (GSACS)	13 ± 3 ab	8 ± 1 b	13 ± 3 ab	24 ± 4 a	58 ± 11	0.08
11	γ-Glutamyl-*S*-methyl-L-cysteine sulfoxide (GSMCS)	43 ± 12 b	29 ± 9 b	69 ± 25 b	166 ± 35 a	308 ± 82	4.05
12	γ-Glutamyl-*S*-(propyl)-L-cysteine	17 ± 3 b	8 ± 1 b	14 ± 4 b	37 ± 7 a	76 ± 15	8.4
	**Total GSAk**	**251 ± 41 b**	**144 ± 21 b**	**339 ± 77 b**	**980 ± 134 a**	**1713 ± 273**	**0.92**
	***S*-alk(en)yl-L-cysteine derivatives (SAk)**						
13	*S*-(2-Propenyl)-allyl-L-cysteine sulfoxide	179 ± 37 a	98 ± 23 a	162 ± 90 a	238 ± 69 a	678 ± 220	-
14	*N*-acetyl-*S*-allyl-L-cysteine sulfoxide (NASACS)	144 ± 35 a	118 ± 45 a	144 ± 40 a	672 ± 391 a	1079 ± 512	-
15	*S*-Butanoyl-L-cysteine sulfoxide (Butiin)	17 ± 3 a	34 ± 16 a	14 ± 3 a	94 ± 28 a	159 ± 50	-
16	*S*-Allyl-L-cysteine (SAC)	302 ± 64 b	348 ± 112 b	391 ± 73 ab	1950 ± 810 a	2991 ± 1059	1.95
17	*N*-acetyl-*S*-allyl-L-cysteine (NASAC)	1.49 ± 0.00 a	10 ± 2 a	3.4 ± 0.1 a	8.6 ± 1.7 a	23 ± 3	-
18	tran*S-S*-(1-Propenyl)-L-cysteine (S1PC)	618 ± 27 a	982 ± 151 a	448 ± 78 a	1862 ± 731 a	3911 ± 987	-
19	*S*-Propyl-L-cysteine (Deoxypropiin)	1417 ± 201 b	1005 ± 106 b	1387 ± 406 b	3226 ± 679 a	7035 ± 1392	-
20	*S*-Allyl-L-cysteine sulfoxide (Alliin)	97 ± 15 a	144 ± 35 a	105 ± 25 a	1037 ± 961 a	1383 ± 1035	1.23
21	tra*N-S*-(1-Propenyl)-L-cysteine sulfoxide (Isoalliin)	1825 ± 629 a	2483 ± 704 a	2059 ± 634 a	8633 ± 7274 a	15,001 ± 9241	-
22	Cycloalliin	120 ± 18 ab	244 ± 42 a	198 ± 47 a	63 ± 15 b	625 ± 122	-
23	*S*-(2-Carboxypropyl)-L-cysteine	382 ± 59 a	275 ± 45 a	389 ± 78 a	7759 ± 7038 a	8804 ± 7220	382.8
24	*S*-Methyl-L-cysteine sulfoxide (Methiin)	2854 ± 763	2203 ± 518	3596 ± 1205	9300 ± 3554	17,954 ± 6040	369.4
25	Methionine sulfoxide	237 ± 29 b	387 ± 61 b	567 ± 94 b	1029 ± 159 a	2221 ± 343	22.1
26	*S*-Propyl-L-cysteine sulfoxide (Propiin)	165 ± 35 b	131 ± 21 b	196 ± 26 b	533 ± 106 a	1024 ± 189	-
27	*S*-Carboxymethyl-L-cysteine (Carbocysteine)	59 ± 8 b	61 ± 9 b	94 ± 19 b	222 ± 41 a	435 ± 78	-
28	*S*-(2-Carboxypropyl)-L-cysteine-glycine	232 ± 79 ab	89 ± 22 b	248 ± 77 ab	472 ± 85 a	1041 ± 264	20.0
29	*S*-Ethyl-L-cysteine sulfoxide (Ethiin)	65 ± 16 b	42 ± 8 b	98 ± 27 b	229 ± 35 a	435 ± 86	-
30	Ajoene	6.6 ± 1.4 b	5.3 ± 0.8 b	12 ± 2 b	31 ± 7 a	55 ± 11	-
31	*N*-acetyl-*S*-(2-carboxypropyl)-L-cysteine (NACPC)	37 ± 5 b	19 ± 2 b	57 ± 8 ab	80 ± 11 a	193 ± 27	-
32	*S*-Allylmercaptoglutathione	1.8 ± 0.4 b	<LOD	1.5 ± 0.1 b	5.0 ± 0.3 a	8.3 ± 0.8	-
33	Allixin	<LOD	13 ± 4 a	19 ± 1 a	9.5 ± 2.a	41 ± 8	-
	**Total SAk**	**8200 ± 1605**	**7810 ± 1506**	**9802 ± 2619**	**36,787 ± 20,673**	**62,599 ± 26,403**	**19.4**
	**Total Organosulfur Compounds**	**8451 ± 1630**	**7953 ± 1518**	**10,141 ± 2690**	**37,767 ± 20,746**	**64,312 ± 26,584**	**12.6**

Results are expressed as mean ± SE (standard error). LOD: Limit of Detection; Different letters (one-way ANOVA) denote significant differences (*p* < 0.05) among the stages (0–2 h; 2–4 h;4–8 h; 8–24 h) for the same compound.

## Data Availability

The data are contained within the article.

## Supplementary Materials

The following supporting information can be downloaded at: https://www.mdpi.com/article/10.3390/antiox12040925/s1, Figure S1: HPLC-HRMS chromatograms of the identified *S*-Alk(en)yl-L-cysteine derivatives in urine after acute intake of Black Garlic.; Figure S2: HPLC-HRMS chromatograms of the identified γ-Glutamyl-*S*-alk(en)yl-L-cysteine derivatives in urine after acute intake of Black Garlic.
