# Supplementary material for: Bioavailability of Organosulfur Compounds after the Ingestion of Black Garlic by Healthy Humans
Source: Antioxidants (Basel). 2023 Apr 13;12(4):925. doi: 10.3390/antiox12040925 (PMC10135770; doi:10.3390/antiox12040925)
Supplement: Supplementary file 1 [file antioxidants-12-00925-s001.zip › antioxidants-2310254-supplementary.pdf]

SUPPLEMENTARY INFORMATION

Bioavailability of Organosulfur Compounds after the Ingestion of Black Garlic by Healthy Humans

Alicia Moreno-Ortega [1,2,*], Gema Pereira-Caro [1,2], Iziar A. Ludwig [3,†], María-José Motilva [3,‡] and José Manuel Moreno-Rojas [1,2,*]

1 Department of Agroindustry and Food Quality, Andalusian Institute of Agricultural and Fisheries Research and Training (IFAPA), Alameda del Obispo, Avda. Menéndez-Pidal, 14004 Córdoba, Spain

2 Foods for Health Group, Maimonides Biomedical Research Institute of Cordoba (IMIBIC), 14004 Córdoba, Spain

3 Agrotecnio Center, XaRTA-TPV, Food Technology Department, Escola Tècnica Superior d'Enginyeria Agrària, University of Lleida, Avda. Alcalde Rovira Roure 191, 25198 Catalonia, Spain

* Correspondence: alicia.moreno.ortega@juntadeandalucia.es (A.M.-O.); josem.moreno.rojas@juntadeandalucia.es (J.M.M.-R.)

† Current address: Departamento de Ciencias de la Alimentación y Fisiología, Facultad de Farmacia y Nutrición, Universidad de Navarra, 31008 Pamplona, Spain.

‡ Current address: Instituto de Ciencias de la Vid y del Vino (ICVV) (Consejo Superior de Investigaciones Científicas-CSIC, Gobierno de La Rioja, UR), Finca "La Grajera", Carretera de Burgos km 6, 26007 La Rioja, Spain.

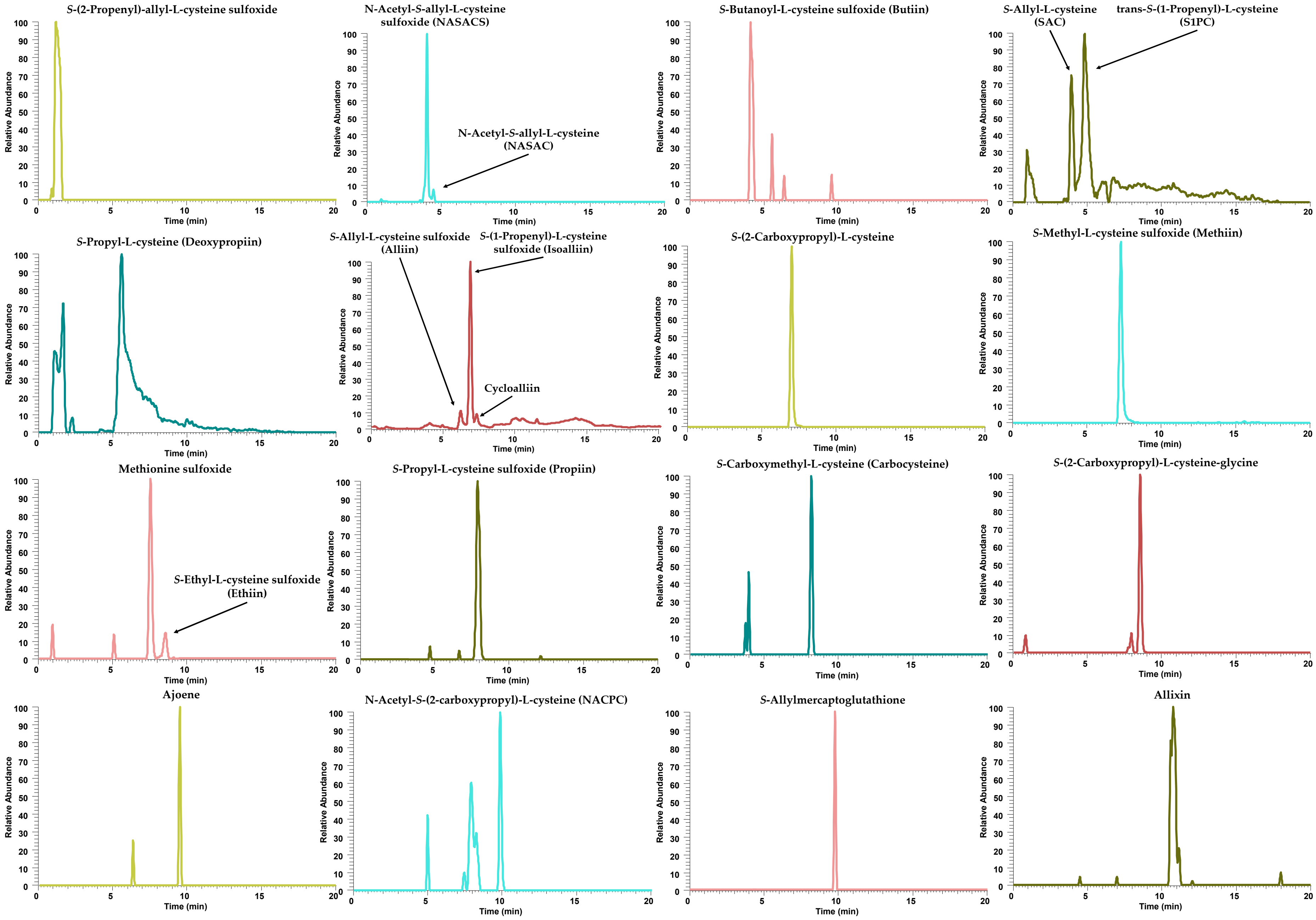

Figure S1. HPLC-HRMS chromatograms of the identified *S*-Alk(en)yl-L-cysteine derivatives in urine after acute intake of Black Garlic.

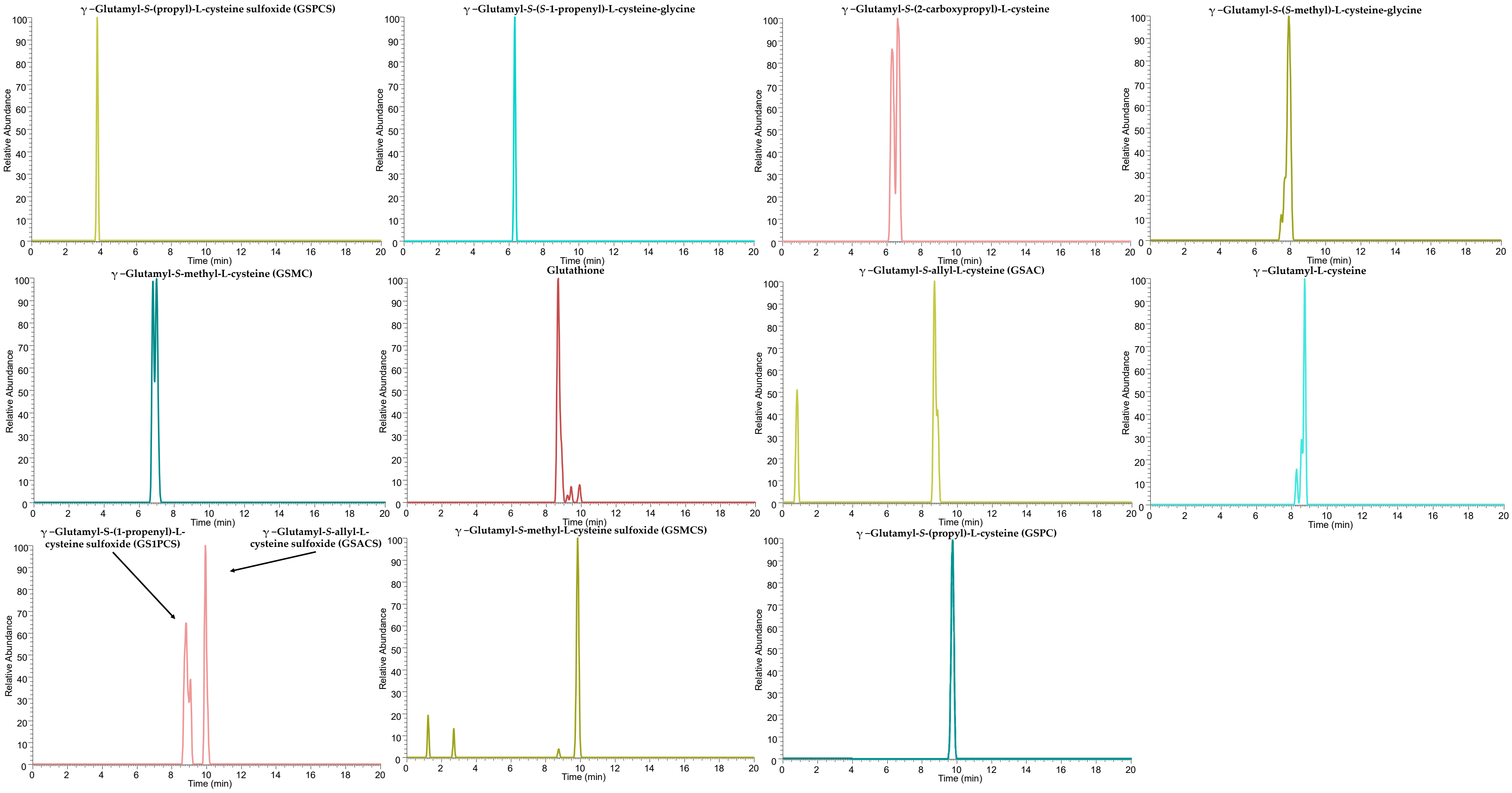

Figure S2. HPLC-HRMS chromatograms of the identified γ-Glutamyl-*S*-alk(en)yl-L-cysteine derivatives in urine after acute intake of Black Garlic.
